# CABG Patients Develop Global DNA Hypermethylation, That Negatively Affect the Mitochondrial Function and Promote Post-Surgical Cognitive Decline: A Proof of Concept in Small Cohort

**DOI:** 10.3390/jcm12124146

**Published:** 2023-06-20

**Authors:** Sri Rahavi Boovarahan, Suresh Babu Kale, Priyanka N. Prem, Sriram Ravindran, Akshayakeerthi Arthanarisami, Jeyashri Rengaraju, Nemat Ali, Senthilkumar Ramalingam, Mohamed Mohany, Abdullah F. AlAsmari, Salim S. Al-Rejaie, Mohammad Waseem, Gino A. Kurian

**Affiliations:** 1 Vascular Biology Laboratory, SASTRA Deemed University, Thanjavur 613401, India; 2 Department of Cardiovascular and Thoracic Surgery, Meenakshi Hospital Tanjore, Thanjavur 613005, India; 3 Department of Pharmacology and Toxicology, College of Pharmacy, King Saud University, P.O. Box 55760, Riyadh 11451, Saudi Arabia; 4 Department of Pharmaceutical Sciences, School of Pharmacy, University of Maryland Eastern Shore, Princess Anne, MD 21853, USA

**Keywords:** mitochondrial function, off-pump CABG, ischemia-reperfusion injury, mild cognitive impairment, peripheral blood mononuclear cells, DNA methylation

## Abstract

Global DNA hypermethylation and mitochondrial dysfunction are reported to be associated with the development of mild cognitive decline (MCI). The present study aims to generate preliminary data that connect the above association with post-surgical coronary artery bypass grafting (CABG) cognitive decline in patients. Data were collected from 70 CABG patients and 25 age-matched controls. Cognitive function was assessed using the Montreal Cognitive Assessment (MOCA) test on day 1 (before surgery) and on the day of discharge. Similarly, blood was collected before and one day after the CABG procedure for mitochondrial functional analysis and expression of DNA methylation genes. Test analysis score suggested 31 (44%) patients had MCI before discharge. These patients showed a significant decrease in complex I activity and an increase in malondialdehyde levels (*p* < 0.001) from the control blood samples. Post-surgical samples showed a significant reduction in blood MT-ND1 mRNA expression from control and from pre-surgical samples (*p* < 0.005), along with elevated DNMT1 gene expression (*p* < 0.047), with an insignificant increase in TET1 and TET3 gene expression. Correlation analysis showed a significant positive relation between cognitive decline and elevated blood DNMT1 and declined blood complex I activity, signifying that cognitive decline experienced by post-surgical CABG patients is associated with increased DNMT1 expression and declined complex I activity. Based on the data, we conclude that both DNA hypermethylation and mitochondrial dysfunction are associated with post-CABG MCI, where the former is negatively correlated, and the latter is positively correlated with post-surgical MCI in CABG cases. Additionally, a multimarker approach that comprises MOCA, DNA methylation, DNMT, and NQR activities can be utilized to stratify the population that is sensitive to developing post-CABG MCI.

## 1. Introduction

Mild Cognitive Impairment (MCI) is a sequence of neural and motor disorders, associated with patients who underwent coronary artery bypass grafting (CABG) [1]. The prevalence rate of MCI is considerably high, affecting more than one-third of cardiac surgery patients [2]. Post-surgical MCI not only compromises the quality of life but may also increase the frequency of re-hospitalization in post-CABG patients [3]. Post-operative cognitive decline after cardiac surgery depends on various factors (modifiable, partly modifiable, and non-modifiable). A few pre/post-operative factors include blood pressure control, glycemic control, creatinine levels, sleep deprivation or sleep apnea, alcohol abuse, neurocognitive reserve, and chronological age [4,5]. Some of the intra-operative factors that can influence cognitive decline includes micro embolism, variation in perfusion pressure, temperature management, arterial pressure management, hemodilution, and anesthesia [6,7]. Evidence from the different studies suggests that, at a cellular level, oxidative stress and inflammation linked to revascularization procedure and alterations in DNA methylation patterns linked to anesthesia promote the development of MCI in patients undergoing surgery [8,9,10].

Reliable MCI diagnosis is often difficult and widely adopted methods include MRI brain imaging for early detection, informant-reported questionnaires, and neurological examination for reflexes [11]. However, these tests have their own limitations with respect to cost-effectiveness in the case of MRI and the lack of experimental evidence in other diagnosis methods including MoCA tests. Not all post-surgical patients experience MCI and hence a simple, cost-effective, reliable blood marker is needed to identify and stratify the sensitive patients. Peripheral blood is easily obtainable, less invasive, less costly, and serves as an ideal sample to monitor systemic changes.

A study of selected participants of Whitehall II imaging supports those changes in peripheral blood DNA methylation are associated with cognitive dysfunction and brain aging [12]. In specific to surgeries, the anesthetics regimen used during the surgery, such as sevoflurane, and propofol can also induce alterations in the DNA methylation levels and their enzymes, which result in cognitive dysfunction [9]. In fact, the niche of both blood cells and cardiac cells may change prior to, during, and after the revascularization procedure, and thereby they are expected to influence the activity of DNA methylating enzymes and subsequent change in levels of DNA methylation. The major change in cellular environments is associated with pro-inflammation and pro-oxidant response from the CABG procedure [13].

The elevated oxidative stress and Ca^2+^ overload in cardiac tissue in response to revascularization injury developed during CABG deteriorates the mitochondrial function. This in turn alters metabolic homeostasis and initiates the inflammatory response via NLRF2 activation, leading to the development of systemic inflammatory response syndrome in bypass surgery cases [14]. Similarly, cell-free mitochondrial DNA fragments act as mitochondrial-linked damage-associated molecular patterns that have been widely associated with high atrial fibrillation rates and mortality observed in off-pump CABG (OPCABG) [15]. Evidence from the literature suggested that peripheral mitochondrial functional perturbation occurs during surgery and is associated with mild cognitive decline, as an outcome of surgical stress [16].

In short, there are high possibilities for mitochondrial functional perturbations and changes in the expression of methylation enzymes in OPCABG patients during the surgery, and accumulating evidence from the literature supports their strong association of such cases with cognitive decline. Considering the fact that no reliable marker to predict MCI with experimental evidence exists for OPCABG patients, in the present study, we hypothesize that testing the expression of DNA methylating enzymes and mitochondrial function along with the MoCA test score can enhance the reliability of MCI identification in OPCABG cases.

## 2. Materials and Methods

### 2.1. Study Design and Participants

A total of 76 elective off-pump CABG patients were enrolled in the study. The eligibility criteria were set as: age group of 40 to 70 years of either gender, absence of any previous heart disease, kidney dysfunction, or neurological disease. Patients with diabetes and hypertension were also included in the study. The anesthesia administered to the patients included propofol, sodium thiopental, midazolam, fentanyl, and vecuronium, and were maintained for a period of 3 to 6 h. The control population comprises 45 healthy individuals without any disease conditions. Approval for the study was provided by the Institutional Ethical Committee (IEC) of Meenakshi Hospital Tanjore, India (Dated 22 November 2017). This study was performed in accordance with the ethical guidelines of the Helsinki Declaration (1975). Written informed consent was provided by all the patients enrolled in this study.

The Montreal Cognitive Assessment Test-Basic (MoCA) was utilized with prior authorization to measure cognition in all individuals according to the instructions, based on socio-demographic characteristics. The test was performed in a controlled atmosphere by well-trained healthcare professionals. The MoCA test was chosen because it was more reliable than the MMSE and had higher sensitivity and specificity [17].

### 2.2. Pre and Post-Surgical Cognitive Assessment

The cognitive function of patients was evaluated using the basic Montreal cognitive assessment (MoCA) test sheet as described previously [18]. The MoCA test was taken by well-trained nursing staff from the patients a day before the OPCABG surgery and on the day of discharge which is typically three days from the date of surgery. As per the test criteria, we have considered a score of <26 as a case of mild cognitive impairment (MCI).

### 2.3. Blood and Tissue Sampling and MPO

The peripheral blood (5 mL) was drawn from the patient before bypass grafting in lithium heparin-coated tubes. Another sample of blood was taken 24 h after the surgery when the patient was in the ICU for routine care. Immediately after collection, the blood was processed for the separation of blood cells and plasma. Myeloperoxidase (MPO) activity was assessed as an inflammatory biomarker in plasma, and malondialdehyde (MDA) levels were estimated in the serum according to the protocols mentioned elsewhere [19,20].

Atrial appendages were collected immediately before and after the OPCABG procedure in seven patients who underwent OPCABG and stored at −80 °C.

### 2.4. Isolation of Mitochondria from Peripheral Blood Mononuclear Cells

The mitochondria from blood were isolated from the peripheral blood mononuclear cells (PBMC) as per the previous procedure with slight modifications [21]. Briefly, the blood was diluted with phosphate-buffered saline (PBS, 1:4) and layered onto density gradient separation media (HiSep™-LSM-1077, HiMedia). The tubes were centrifuged at 2000 rpm for 20 min at 10 °C without brakes to separate the mononuclear cells from RBC and plasma. Plasma was used immediately for RNA isolation and ATP estimation. The PBMC fraction was collected and washed with PBS. The solution was then centrifuged at 5000 rpm for 5 min at 4 °C to pellet the PBMC and stored at −80 °C after discarding the supernatant. To the leukocyte pellet, 200 μL of isolation buffer (IB-0.25 M sucrose and 10 mM HEPES, pH = 7.5) was added and frozen. To separate the mitochondria from the cells, five cycles of repeated freeze-thaw were carried out after the addition of IB, and the mixture was centrifuged at 1000× *g* for 10 min at 4 °C to separate broken cells. The supernatant containing mitochondria was separated and centrifuged at 20,000× *g* to obtain a mitochondrial pellet. Oxygraph (Hansatech, Pentney, UK) was used to assess the quality of mitochondria by respirometry.

### 2.5. Mitochondrial MT-ND1 Expression by Real-Time PCR

The total RNA was isolated from the plasma using TRIzol™ reagent as per the instructions in the kit (#15596026, Thermo Fischer Scientific, Waltham, MA, USA). The cDNA conversion was carried out using the Verso cDNA synthesis kit (#AB1453A, Thermo Fischer Scientific, USA). The mitochondrial ND1 primers were designed to amplify the gene of interest using SYBR green chemistry. The PCR analysis was completed in real-time using the Applied Biosystems™ 7500 with ROX reference dye. The expression was normalized with GAPDH as a control. The gene expression was calculated by the method of Livak [22].

### 2.6. Complex I Activity Assay

The analysis of mitochondrial complex I was completed as per the previously described procedure [23]. Briefly, the mitochondrial pellet was lysed by giving an osmotic shock in a hypotonic medium (25 mM K_2_PO_4_, 5 mM MgCl_2_ pH 7.2). To 25 µg of mitochondrial protein, 200 µL of reaction buffer (50 mM Tris, 0.8 mM NADH, 240 µM KCN, and 4 µM antimycin A pH = 8.0) was added. The reaction was initiated with the addition of 50 µM decylubiquinone. For 5 min, the reaction was monitored at 340 nm using a spectrophotometer with the temperature maintained at 37 °C (Synergy H1, BioTek, Winooski, VT, USA). The rotenone-insensitive activity was estimated after the addition of 4 µM rotenone and the monitoring was continued for an additional 3 min. The enzyme activity was expressed as nM/min/mg protein.

### 2.7. Adenosine Triphosphate (ATP) Measurement

The separated plasma was immediately treated with a lysis buffer containing an inhibitor of endogenous ATPase and shaken for 10 min. A substrate buffer, 100 μL, was then added, and incubated in the dark for 10 min and the luminescence intensity was recorded as per the kit instructions (ATP lite, PerkinElmer, Waltham, MA, USA) using Synergy H1 Multimode reader (BioTek, Shoreline, WA, USA). The standard curve for ATP was drawn from 10 μM to below for an estimation of the ATP level in the sample.

### 2.8. Mitochondrial DNA (MtDNA) Copy Number Measurement

The mitochondrial-DNA copy number (mt-CN) was estimated from the peripheral blood using the protocol of Zhou et al. [24]. In brief, 1 mL of blood was used for extraction of genomic DNA, and the expression of mitochondrial-encoded ND1 and nuclear-encoded HGB were assessed using qPCR (ABI 7500, Applied Biosystems, Waltham, MA, USA). Each sample was run in triplicates for amplification and quantification using SYBR green chemistry (#F415S, Thermo Scientific, USA). A standard curve was constructed using calibrator DNA obtained from healthy controls and used for comparison. The ratio of the ND1 copy number and HGB copy number was taken for each sample using a standard curve, and the value was given as mt-CN.

### 2.9. Methylation Analysis

The total RNA was isolated from the tissue using TRIzol™ reagent as per the kit’s instructions (#15596026, Thermo Fischer Scientific, USA). The cDNA conversion was carried out using the Verso cDNA synthesis kit (#AB1453A, Thermo Fischer Scientific, USA). The DNMT1, DNMT3A, DNMT3B, TET1, TET2, TET3, and GAPDH primers were designed to amplify the genes of interest using SYBR green. The primer sequence details are presented in Table 1.

The PCR analysis was completed in real-time using the Applied Biosystems™ 7500 instrument with ROX reference dye. The expression was normalized with GAPDH as a control, and the relative gene expression was calculated by the method of Livak [22]. DNA was isolated following the HiMedia kit protocol, and global DNA methylation was evaluated in the heart DNA using MethylFlash™ Global DNA Methylation (5-mC) ELISA Easy Kit (Epigentek).

### 2.10. Statistical Analysis

According to the test criteria, a patient was categorized as MCI if the overall score after evaluation of 10 cognitive domains, adjusted for education, was less than 26. As a result, if the student’s education was less than four years, one point was added to the overall exam score, and if the student was illiterate, one point was deducted. In addition to the correlation of internal measures with the composite Z-score, Cronbach’s alpha value was calculated for internal validation of the MoCA test sheet. Graph Pad Prism 7.0 was used to analyze the data, which was expressed as mean ± SEM. The *t*-test and two-way ANOVA were used to compare the significant differences between the groups. Pearson’s coefficient correlation was used to do the correlation study. Statistical significance was defined as a *p*-value of less than 0.05.

## 3. Results

### 3.1. Patients Characteristics

**Figure 1:**
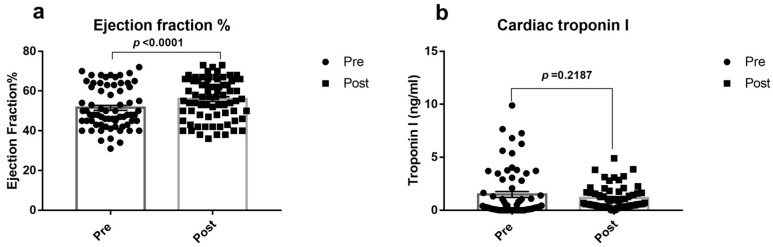
OPCABG surgical outcome: (**a**) Percentage ejection fraction and (**b**) Plasma cardiac troponin I level are measured in off-pump CABG cases before and 24 h after surgery. The graph is presented as mean ± SEM with individual observations. *p* < 0.05 was considered statistically significant.

The study was performed between December 2017 and December 2018 in collaboration with the Department of Cardiothoracic and Vascular Surgery, Meenakshi Hospital, Tanjore, India on 22 November 2017. Of the 76 patients screened as per the eligibility criteria, only 70 were included in the study. The rest of them were excluded, in case the surgery went on-pump due to intraoperative complications or due to non-compliance of the patient to complete the cognitive testing. The left ventricular ejection fraction was improved while cardiac troponin I decreased in patients after surgery from the pre-surgical level (Figure 1), indicating a successful surgery.

The baseline characteristics of the overall study population are presented in Table 2.

### 3.2. Cognitive Testing and Impairment

All the 70 cases evaluated in the study were extubated within 24 h after grafting. There were no cases of reintubation or death on the day of discharge. The MoCA test sheets were validated for internal consistency and Cronbach’s alpha value was 0.936. All the 10 internal measures of the test sheet were correlated with the composite z-score for the population and 8/9 measures showed a significant correlation (Table 3). The control population did not show any cognitive decline.

**Figure 2:**
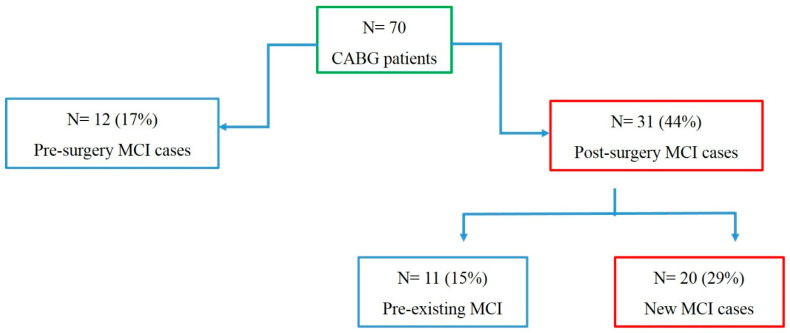
MCI cases: Flow chart representing the number of cases having a decline in MoCA score post-surgery compared to their pre-surgery values among the entire population.

According to our MoCA score data, out of 70 OPCABG cases, 12 patients showed a MoCA score < 26 before surgery (17% of the total population), and out of 12, one patient became normal (MoCA score > 26) post-surgery. Thirty-one patients showed a MoCA score < 26 after surgery (44% of total subjects), denoting post-surgical MCI. Out of 31 post-surgery MCI patients, 20 subjects (29% of the total subjects) recorded low MoCA test scores from their pre-surgical test scores (MoCA > 26) (Figure 2), indicating newly formed MCI upon surgery.

Diabetes and blood pressure are predisposing factors for vascular and cognitive impairment and across the new post-surgical MCI population, we found that most patients had diabetes (40%) or hypertension (10%) or both (40%) as risk factors. However, both the pre-surgical MCI and post-surgical MCI cases did not show any correlation with their respective cTnI values (Table 4).

### 3.3. Blood DNA Methylation Analysis

Blood DNA methylation analysis of these 20 newly formed MCI cases, according to MoCA test score, showed that post-surgical samples of these patients showed a significant upregulation in their DNMT1 (*p* = 0.0393) and TET3 (*p* = 0.0056) gene expression from the control population (Table 5). Correlation analysis of post-surgical DNMT1 expression of the MCI population with their MoCA score showed a significant negative correlation, suggesting the strong influence of methylation over MCI (r = −0.8573, *p* = 0.0498).

**Figure 3:**
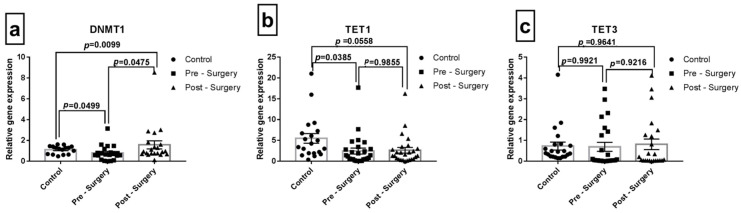
Gene expression analysis of Blood DNA methylation enzymes: (**a**) DNMT1 (Control: 1.04 ± 2.90, Pre: 0.67 ± 0.15, Post: 1.57 ± 0.34, (**b**) TET1 (Control: 5.48 ± 1.22, Pre: 2.44 ± 0.47, Post: 2.62 ± 0.51, (**c**) TET3 (Control: 0.72 ± 0.15, Pre: 0.68 ± 0.14, Post: 0.80 ± 0.16). Data are plotted as individual points with mean ± SEM. *p* < 0.05 was considered significant.

In the overall population (*n* = 70), according to Figure 3, among the different DNA methylating genes, DNMT1 gene expression was significantly (*p* = 0.0475) altered in the post-OPCABG sample and found to be high from the pre-surgical blood samples.

### 3.4. Blood Mitochondrial Functional Assessment

Table 6 represents the summary of the mitochondrial functional assessment parameters and MPO activity of the newly formed MCI cases *(n* = 20). The mitochondrial copy number, ATP levels, and MPO enzyme were significantly higher in both the pre-surgical and post-surgical samples of the MCI cases from the control, while the complex I activity declined significantly in these samples (Table 6). However, no significant differences were noted in the above parameters between pre- and post-surgical samples of the MCI cases. A correlation study of these parameters with post-surgical MoCA scores of MCI cases showed a strong positive correlation with complex I activity (r = 0.8652, *p* = 0.0398) and a negative correlation with the mitochondrial copy number (r = −0.8309; *p* = 0.0489), suggesting the influence of mitochondrial functional status on the cognitive function. Similar observations were observed with MPO, and the antioxidant enzyme as well, where pre- and post-surgical samples showed a significant increase in their activity from the control.

**Figure 4:**
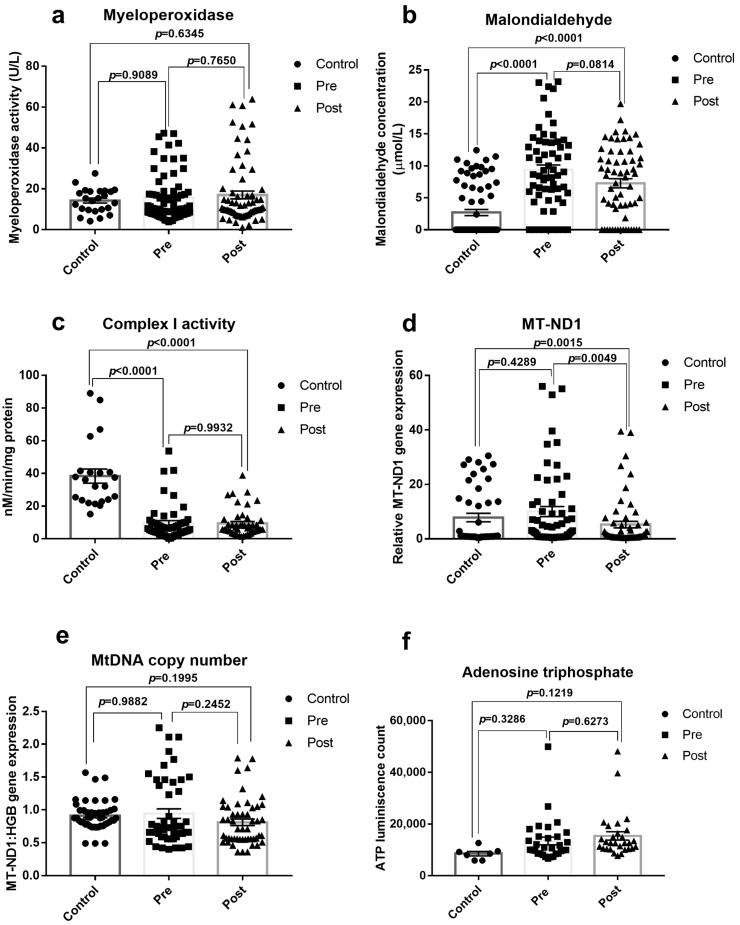
Blood mitochondrial function and oxidative stress assessment: The mitochondrial function and oxidative stress were measured in off-pump CABG cases before and 24 h after surgery along with the level from the healthy control population: (**a**) Myeloperoxidase activity, expressed in U/L (Control: 14.22 ± 2.90, Pre: 15.47 ± 1.82, Post: 16.98 ± 2.05), (**b**) Serum malondialdehyde concentration, expressed in µmol/L (Control: 2.70 ± 0.31, Pre: 9.34 ± 1.19, Post: 7.27 ± 0.93), (**c**) The complex I activity of peripheral blood mononuclear cells mitochondria, expressed in nM/min/mg protein (Control: 40.01 ± 8.94, Pre: 9.48 ± 1.32, Post: 8.96 ± 1.33), (**d**) MT-ND1 relative expression in plasma (Control: 7.84 ± 1.96, Pre: 10.03 ± 1.29, Post: 5.23 ± 0.68), (**e**) Blood mtDNA copy number (Control: 0.91 ± 0.13, Pre: 0.94 ± 0.13, Post: 0.81 ± 0.11), (**f**) Plasma ATP level, expressed as ATP luminescence count (Control: 8557.01 ± 2334.24, Pre: 13,450.90 ± 2415.85, Post: 15,394.38 ± 2858.66). The graph is presented as a mean ± SEM with individual observations. *p* < 0.05 was considered statistically significant.

However, when considering the overall population consisting of all the MCI and non-MCI cases, there were no significant changes observed with MPO, Mt copy no, and ATP from the control population unlike the MCI cases (Figure 4a,e,f). However, the significant changes in complex I activity in MCI cases were prevalent in the overall population as well (Figure 4c). Incidentally, malondialdehyde, the lipid peroxidation byproduct was found to be significantly increased across the pre- and post-surgical samples from control, when considering the overall OPCABG population.

### 3.5. Cardiac Tissue Analysis

In order to understand the relationship between blood-borne changes and myocardial tissue, we analyzed the atrial appendages of 20 MCI patients. However, the quality of the tissue obtained in a few patients was not good and thus not included in the study. In the present study, we included only seven patient samples obtained before and after surgery, which is of sufficient quantity and quality. As shown in Table 7, among the DNA methylation enzymes, only DNMT1 showed higher mRNA expression in the atrial appendage by 2.6 folds after the OPCABG procedure, while denovo methylating enzymes DNMT3A (2^∆CT^ 1.21 ± 0.12 vs. 1.24 ± 0.21) and DNMT 3B (2^∆CT^ 0.88 ± 0.09 vs. 0.94 ± 0.31) in post-surgical samples did not show any significant differences when compared with the pre-surgical tissue. Since the increase in DNMT1 expression level can influence and increase the overall cardiac DNA methylation level, the Global DNA methylation level of pre- and post-surgical atrial appendages was measured, and we found no significant post-surgery changes in %5 mC level (*p*-value = 0.2793).

Hence, we evaluated the mRNA expression of demethylating enzymes TETs involved in the DNA demethylation process in atrial appendages obtained from OPCABG patients before and after the procedure. Among the methylation erasers, TET1 and TET3 gene expression were higher in post-surgical atrial appendages by 2.2 (*p* = 0.04) and 2.75 folds (*p* = 0.04), respectively from pre-surgical atrial samples. However, TET2 mRNA did not significantly differ in expression upon surgery (2^∆CT^ 0.13 ± 0.06 vs. 0.12 ± 0.03).

To check if these changes in the mRNA expression of methylation enzymes influence the outcome of surgery, we made a correlation analysis of mRNA expression of DNMTs and TETs post-surgery with the ejection fraction and troponin I post-surgery. We found a negative correlation of DNMT1 (r = −0.885, *p* = 0.046) and TET1 (r = −0.849, *p* = 0.033) post-surgery with post EF%. However, no correlation was found between DNMT1 with TnI post-surgery. However, TET1 expression negatively correlated with post-surgery TnI values (r = −0.942, *p* = 0.017).

**Figure 5:**
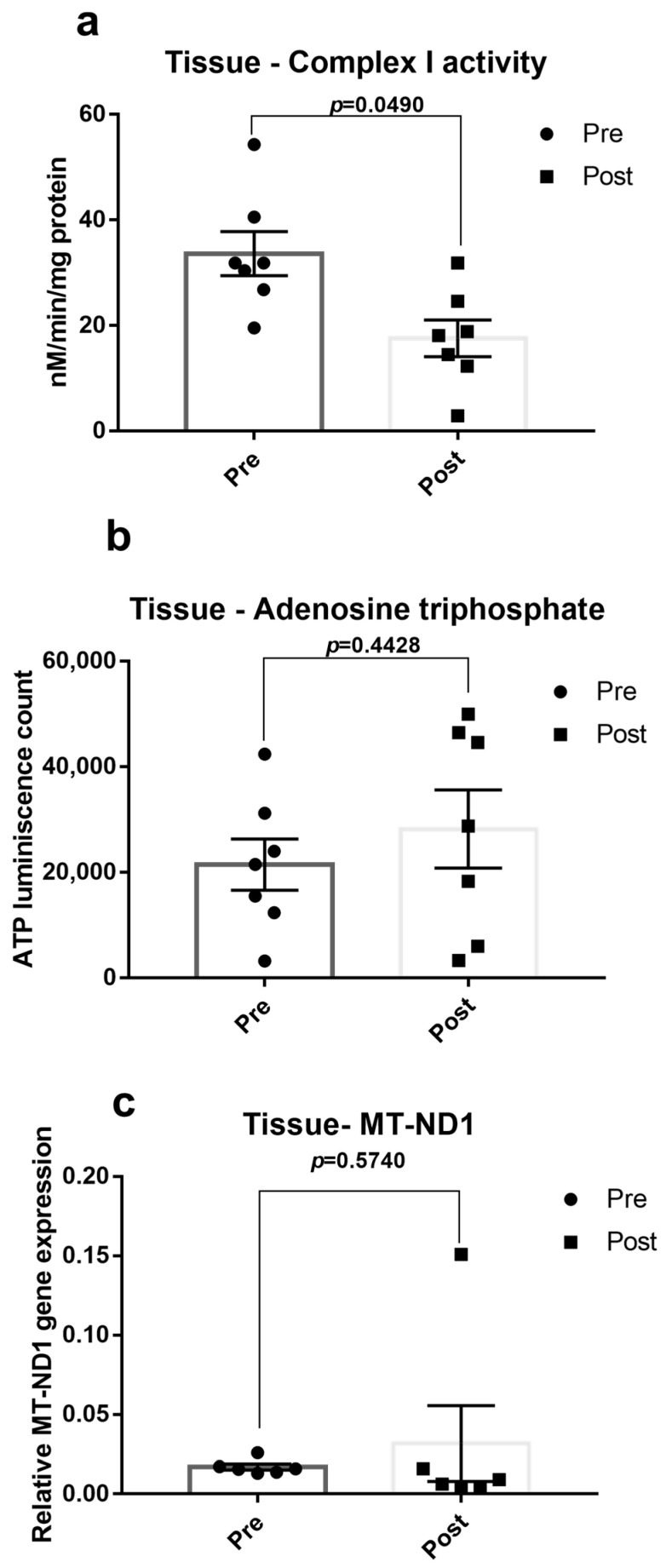
Atrial tissue mitochondrial function: (**a**) The complex I activity of mitochondria, (**b**) ATP level, and (**c**) MT-ND1 expression were measured in seven off-pump CABG cases atrial appendages before and 24 h after surgery. The graph is presented as a mean ± SEM with individual observations. *p* < 0.05 was considered statistically significant.

To understand the relationship between the mitochondria in the heart and blood, we measured the mitochondrial parameters in atrial appendages from seven OPCABG patients. In both post-blood and atrial appendage OPCABG samples, complex I activity was low, and ATP level was high upon surgery (Figure 5a,b). However, in the case of the MT-ND1 gene, the expression was high in the post-operative atrial appendages, unlike the blood sample that showed low gene expression (Figure 5c).

## 4. Discussion

The evaluation of cognitive function in patients after the OPCABG procedure is imperative due to the increased incidence of post-surgical MCI. OPCABG patients often experience stress from anesthetic regimens as well as surgical procedures that may lead to the development or progression of MCI. However, the development of MCI in post-surgical OPCABG patients may take varying amounts of time depending on the severity of injury or abnormalities that occur during surgery. MCI is a progressive disorder, where it can have a long-lasting impact and the pathology starts at a cellular level. Though many questionnaire-based tests are available to evaluate high-order cognitive function in humans, there is no consensus on the clinically relevant tests that should be adopted due to multiple reasons [25], of which lack of experimental evidence is a critical factor. Thus, in the present study, apart from the utilization of questionnaire-based surveys to assess cognitive decline in OPCABG patients, we generated experimental evidence with respect to DNA methylation and mitochondrial function in blood and tissue to improve the reliability of the MoCA test. Montreal Cognitive Assessment (MoCA) was used for the present study where the test covers 10 cognitive domains and takes less than 10 min for the completion of the survey [18]. Accordingly, we found a declined MoCA score by 17% in the pre-surgical patients, and at the discharge of post-surgical patients, the decline was around 44%, of which 29% of the patients were newly identified MCI subjects, indicating the causative influence of surgery.

Further, we noted a significant increase in the blood DNMT1 expression (involved in DNA methylation maintenance) in 61% of the post-OPCABG patients who had low MoCA scores (44%). However, both the pre- and post-OPCABG patient samples showed a significant increase in DNMT1 expression from the control (41% and 54% of the population respectively). These observations suggest that the surgical procedure had a prominent influence on the epigenetic alterations that may control the expression of a few genes involved in the aftereffects of post-surgical OPCABG patients.

DNA methylation mediated via DNMT1, DNMT3A, and DNMT3B play a critical role in neural plasticity via inducing CpG methylation [26]. Studies have also reported that brain function, such as synaptic plasticity, memory, and learning can be changed with modified transcriptome, regulated via epigenetic alterations, such as DNA methylation, histone modification, and nucleosome remodeling [27]. Few investigators have shown a change in blood DNA methylation that can sense cognitive dysfunction [12] and associated cardiovascular disease pathology [28]. A recent study has demonstrated an elevated DNMT1 expression during cardiac revascularization protocol, where the latter procedure is adopted in CABG [29]. A study by Caputi et al. in 2021 proved that changes in DNA methylation and gene expression may occur as a response to surgical stress, rather than to anesthesia exposure in breast surgery patients [30]. On the other hand, anesthetic drugs may also induce changes in DNA methylation via the regulation of different DNMT subgroups’ gene expression and their activity. For instance, in recent preclinical studies, sevoflurane and isoflurane anesthetics were reported to upregulate the DNMT3A, 3B, and DNMT1 gene expression respectively, resulting in hypermethylation of BDNF and Reelin genes, resulting in synaptic plasticity deficiency and thereby leads to cognitive impairment [31,32]. Additionally, studies have shown that postoperative global hypomethylation of leukocyte DNA due to anesthesia was associated with cognitive impairment in a cohort of 124 hip surgery patients [9]. On the contrary, propofol and isoflurane anesthetics were found to unalter the leukocyte DNA methylation according to a recent study [33], suggesting that more studies are required in this direction to unfold the underlying mechanism behind the role of anesthesia on the surgery-induced DNMT changes and consecutive MCI. However among the five different anesthetics (propofol, sodium thiopental, midazolam, fentanyl, vecuronium) used in the present study for the surgery, three anesthetics propofol [34], midazolam [35] and fentanyl [36] are reported to alter methylation level and its gene expression, therefore this 61% increase in methylation in post-surgical MCI cases may be the cumulative impact of anesthesia and surgery. However, no significant correlation was observed between the specific anesthesia, methylation, and MoCA score in the present study.

A recent study has reported that mitochondrial dysfunction is a major contributor to the manifestation of cognitive impairment and neuronal death observed in neurodegenerative diseases. MCI is associated with mitochondrial dysfunction in blood lymphocytes by decreasing mitochondrial ATP production and increasing proton leak and oxidative stress, thereby acting as a potential biomarker in the progression of neurodegeneration [37]. The surgical stress response can trigger pro-inflammation and pro-oxidant generation that can lead to cellular and subcellular damage. In both events, mitochondria play a critical role as a triggering source and act as the target site. Mitochondria present in heart tissue is involved in energy production and regulates vital cellular functions like calcium homeostasis, ROS signaling, and cell death pathway. In fact, cardiac mitochondria were reported to be dysfunctional in cardiac surgeries that include CABG procedures [38]. Previous studies have shown that these dysfunctional mitochondria can promote multiple organ failure that can even lead to the mortality of patients in critical cases [39,40].

In the present study, we noted a significant decline in ND1 expression along with a corresponding decline in complex I activity in the blood from OPCABG-assigned patients (Post-surgery) from the normal control, showing mitochondrial function impairment. Mitochondrial copy number and ATP were also found to be elevated in OPCABG patients from the normal control. However, the higher magnitude of changes due to the surgery was observed only with the significant change in MT-ND1 expression between the pre- and post-samples (*p* = 0.0315) (Figure 4). However, in patients who had low MoCA scores post-surgery from control, all the measured mitochondrial parameters (MT-ND1, NQR, Mt copy no., ATP) were found to be further altered with a higher significance (Table 6).

Few preclinical studies have shown that the cells under shear stress can evoke ATP release via endothelial cells [41] into the blood. Hadem and his colleagues reported endothelial dysfunction following the CABG procedure [42]. Moreover, elevated ATP in circulation is linked to systemic inflammatory responses and acts as damage-associated molecular patterns that can trigger the development of multiple organ abnormalities. It is well known that the CABG procedure can induce shear stress in patients, and are expected to have a high level of circulatory ATP. Accordingly, in the present study, we found an elevated level of circulatory ATP in patients before and after surgery when compared with healthy control (Figure 4f). This rise in circulatory ATP (comparatively higher after surgery) and mitochondrial dysfunction did impart elevated inflammatory responses and oxidative stress (as we expected), measured via a significant change in myeloperoxidase activity and blood lipid peroxidation levels in the blood after surgery, especially in MCI cases from control (Table 6, Figure 4a,b). Many studies in the literature emphasized that oxidative stress can modulate DNA methylation by oxidizing DNA, increasing TET-mediated hydroxymethylation, and influencing the produce the methyl donor S-adenosylmethionine by interfering with the binding of DNA methyltransferases [43]. A recent study has shown that hypermethylation at the CREBBP gene is associated with cognitive impairment in 551 participants from Mexican American cohort [44]. In fact, CREB activation is induced after ROS and is critical for the cell survival of neurons. Dysfunctional or damaged mitochondria that can act as a source for ROS generation are found to be involved in cognitive decline. A recent study has shown that Mfn2 expression in the brain can be modified by DNA methylation and can play a critical role in repeated mild traumatic brain injuries-induced persistent cognitive deficits [45]. Indeed, there is much MRI evidence available in support of perioperative brain injury and its potential for long-term dementia.

Despite a significant improvement in the ejection fraction and reduction in cardiac injury marker troponin I in patients before discharge (Figure 1), mitochondrial dysfunction, the index of cellular dyshomeostasis, and CABG-associated DNA Methylation gene expression changes persisted in the blood of OPCABG patients of the present study. To understand the relationship between the surgery-associated mitochondrial and DNA methylation gene expression changes in the heart and blood, we measured the mitochondrial parameters in atrial appendages from seven OPCABG patients. In both post-blood and atrial appendage OPCABG samples, complex I activity was low, and ATP level was high upon surgery (Figure 5a,b) along with elevated DNMT1 expression. However, in the case of the MT-ND1 gene, the expression was high in the post-operative atrial appendages, unlike the blood sample that showed low gene expression (Figure 5c).

## 5. Conclusions

OPCABG procedure imparts mild MCI in 29% of CVD patients, measured via MoCA test. These OPCABG patients showed corresponding significant changes in DNMT1 gene expression and mitochondrial dysfunction in blood. Besides, the changes in the blood were in correlation to the atrial sample collected before and after the OPCABG procedure. This outcome of an increase in DNMT1 and mitochondrial dysfunction in the myocardium contributes to increased inflammation and oxidative stress in blood along with the presence of mitochondrial-dependent DAMP in blood. Collectively, the MoCA test along with a significant change in DNMT1 and mitochondrial functional activity especially via complex I activity can be a useful measure to predict the cognitive changes (that account for the anesthesia and surgical stress) linked to the revascularization procedure adopted in OPCABG.

## Figures and Tables

**Table 1:** Primer details: The forward and reverse primer sequences for the DNA methylation and mitochondrial functional genes are presented.

Gene	Forward Primer	Reverse Primer
DNMT1	5′-GGCTCCGTTCCATCCTTCTG-3′	5′-CAAATCTTTGAGCCGCCTGC-3′
DNMT3A	5′-GGGGACGTCCGCAGCGTCACAC-3′	5′-CAGGGTTGGACTCGAGAAATCGC-3′
DNMT3B	5′-CCTGCTAATTACTCACGCCCC-3′	5′-GTCTGTGTAGTGCACAGGAAAGCC-3′
TET1	5′-TCCTGGTGCTATTCCAGTCC-3′	5′-CAGGAAGGAAGACAGGCAAG-3′
TET2	5′-ACTCACCCATCGCATACCTC-3′	5′-TCAGCATCATCAGCATCACA-3′
TET3	5′-CCCAGACTCCAACTGCTAC-3′	5′-TGGGTCCTCCATTCTGAGAC-3′
GAPDH	5′-CCTGCACCACCAACTGCTTA-3′	5′-GGCCATCCACAGTCTTCTGA-3′
MT-ND1	5′-AACATACCCATGGCCAACCT-3′	5′-AGCGAAGGGTTGTAGTAGCCC-3′
Human β globin	5′-GAAGAGCCAAGGACAGGTAC-3′	5′-CAACTTCATCCACGTTCACC-3′

**Table 2:** Baseline characteristics of patients undergoing off-pump CABG surgery: The baseline characteristics of patients undergoing OPCABG are presented. The values in brackets present the percentage of the population.

Characteristics	Off-Pump CABG Patients (*n* = 76)
Age-years	57.61 ± 7.19
Male sex-no. (%)	60 (79)
Clinical history-no. (%)	
Diabetes	53 (70)
Hypertension	33 (43)
Smoker	07 (9)
Alcoholic	09 (12)
Left ventricular ejection fraction-no (%)	
Grade 1 (≥50%)	37 (49)
Grade 2 (35 to 49%)	35 (46)
Grade 3 (20 to 34%)	04 (05)
Grade 4 (<20%)	00 (00)
Disease vessel—no. (%)	
Triple	72 (94)
Double	02 (03)
Single	02 (03)
Education—no. (%)	
More than 10 years	17 (22)
4 years to 10 years	56 (74)
Illiterate	03 (04)
Ejection fraction %	Pre-surgery: 51.03 ± 0.04 Post-surgery: 56.06 ± 0.04
cTnI (ng/mL)	Pre-surgery: 1.50 ± 0.02 Post-surgery: 1.15 ± 0.01

**Table 3:** Correlation of individual measures of the MoCA test with composite z-score: A composite score was calculated by averaging all z-scores of the nine primary measures from the MoCA test sheet. A *p*-value of < 0.05 was considered statistically significant.

Function Tested	Pre-Surgery		Post-Surgery	
	r Value	*p* Value	r Value	*p* Value
Executive function	−0.03	0.775	0.091	0.459
Fluency	0.35	0.003	0.35	0.003
Orientation	0.70	<0.0001	0.70	<0.0001
Calculation	0.43	0.0002	0.35	0.003
Abstraction	0.64	<0.0001	0.64	<0.0001
Delayed Recall	0.71	<0.0001	0.69	<0.0001
Visuo-perception	0.72	<0.0001	0.63	<0.0001
Naming	0.62	<0.0001	0.48	<0.0001
Attention	0.00	-	0.00	-
Total score	0.96	<0.0001	0.97	<0.0001

**Table 4:** Cardiac troponin-I changes in population with mild cognitive impairment: The significance level for paired *t*-test was set at *p* < 0.05. Pearson’s correlation coefficient (r) is presented along with the significance value in brackets.

Characteristic	*p* Value	r Value
cTnI in cases with pre-surgery MCI	0.469	0.028 (*p* = 0.891)
cTnI in cases with post-surgery MCI	0.463	−0.072 (*p* = 0.7200)

**Table 5:** mRNA expression changes of the DNA methylating enzymes genes in post-surgical MCI cases: The changes in the gene expression of control, pre-surgery and post-surgery groups, normalized with GAPDH genes are presented for the post-surgical MCI patients (*n* = 20). The *p* values of the comparisons between the groups (*t*-test) are also presented. Pearson’s correlation of post-surgical DNMT and TET gene expression values with their corresponding MoCA scores for the same cases have been calculated and the corresponding *p*-value and r value are presented.

Genes	Fold Changes in Gene Expression			Unpaired *t* Test—*p* Value			Post Surgery Correlation (vs. MoCA Score): Pearson’s Coefficient	
	Control	Pre-Surgery	Post-Surgery	Ctrl vs. Pre	Ctrl vs. Post	Pre vs. Post	r Value	*p* Value
DNMT1	1.11 ± 0.09	2.62 ± 0.90	3.08 ± 1.89	0.0171	0.0393	0.8186	−0.8573	0.0498
DNMT3A	1.32 ± 0.18	1.37 ± 0.14	1.38 ± 0.16	0.9181	0.9542	0.9674	−0.5698	0.1780
DNMT3B	1.43 ± 0.04	1.4 ± 0.44	1.28 ± 0.16	0.8452	0.8903	0.8902	−0.7890	0.4298
TET1	5.27 ± 1.12	2.06 ± 1.03	4.21 ± 2.24	0.1301	0.6528	0.4030	0.7803	0.7420
TET2	1.23 ± 0.21	0.81 ± 0.09	0.79 ± 0.08	0.5231	0.7520	0.9451	0.6931	0.5077
TET3	0.74 ± 0.18	1.15 ± 0.42	5.43 ± 2.88	0.3189	0.0056	0.1684	0.6734	0.3591

*p* < 0.05 was considered statistically significant.

**Table 6:** Mitochondrial function and oxidative stress analysis in post-surgical MCI cases: The changes in the mitochondrial parameters of control, pre-surgery and post-surgery groups, are presented for the post-surgical MCI patients (*n* = 20). The *p* values of the comparisons between the groups (*t*-test) are also presented. Pearson’s correlation of post-surgical values with their corresponding MoCA scores for the same cases has been calculated and the corresponding *p*-value and r value are presented.

Mitochondrial Parameters	Control	Pre-Surgery	Post-Surgery	Unpaired *t* Test—*p* Value			Post Surgery Correlation (vs. MoCA Score): Pearson’s Coefficient	
				Ctrl vs. Pre	Ctrl vs. Post	Pre vs. Post	r Value	*p*-Value
ND1 expression (Relative ND1 expression)	4.34 ± 1.18	8.91 ± 3.12	2.46 ± 0.98	0.752	0.2227	0.1264	0.8971	0.5268
NQR activity (nM NADH oxidized/min/mg protein)	38.34 ± 4.33	5.62 ± 1.29	4.76 ± 0.95	<0.0001	<0.0001	0.6331	0.8652	0.0398
Mt Copy no.	0.91 ± 0.03	1.55 ± 0.62	1.38 ± 0.37	0.0165	0.0106	0.806	−0.8309	0.0489
ATP (Luminescence counts)	4997.26 ± 1362.41	15,776.25 ± 4962.66	17,299.42 ± 4192.17	0.0009	<0.0001	0.8212	−0.7043	0.1823
MPO activity (U/L)	14.22 ± 1.21	20.1 ± 4.30	22.63 ± 4.30	0.0125	0.0263	0.8337	0.7028	0.5621

*p* < 0.05 was considered statistically significant. ND1: NADH dehydrogenase subunit 1; NQR: NADH Ubiquinone Oxidoreductase; ATP: Adenosine triphosphate; MPO: Myeloperoxidase

**Table 7:** Cardiac tissue DNA methylation analysis: The gene expression data are presented as fold changes of the pre- and post-surgical samples’ genes from GAPDH with their *p* values. Global DNA methylation was measured as %5 mC, * *p* < 0.05 vs. Pre-surgery. Further, Pearson’s coefficient r value and *p*-value of correlation analysis of the gene expression with %EF and TnI are presented.

	Pre-Surgery Mean ± SEM	Post-Surgery Mean ± SEM	*p*-Value	Post Surgery Correlation: Pearson’s Coefficient r Value—(vs. %EF)	Post Surgery Correlation: *p* Value—(vs %EF)	Post Surgery Correlation: Pearson’s Coefficient r Value—(vs. TnI)	Post Surgery Correlation: *p* Value—(vs. TnI)
mRNA expression: DNMT1	0.17 ± 0.02	0.44 ± 0.11 *	0.04	−0.885	0.046	−0.846	0.154
mRNA expression: DNMT3A	1.21 ± 0.12	1.24 ± 0.21	0.89	−0.121	0.812	0.212	0.368
mRNA expression: DNMT3B	0.88 ± 0.09	0.94 ± 0.31	0.85	−0.113	0.561	0.131	0.412
mRNA expression: TET1	2.61 ± 0.63	5.65 ± 2.88 *	0.04	−0.849	0.033	−0.942	0.017
mRNA expression: TET2	0.13 ± 0.06	0.12 ± 0.03 *	0.99	−0.212	0.312	0.134	0.381
mRNA expression: TET3	0.08 ± 0.03	0.22 ± 0.11	0.04	−0.293	0.931	0.463	0.433
Global %5 mC	2.64 ± 0.40	2.51 ± 0.29	0.28	0.413	0.180	0.708	0.180

## Data Availability

The datasets generated during and/or analysed during the current study are available from the corresponding author on reasonable request due to privacy reasons.
